# PD11 First Comprehensive Assessment Of Artificial Intelligence At Canada’s Drug Agency: Evidence Review Methods, Lessons Learned, And Next Steps

**DOI:** 10.1017/S0266462325101487

**Published:** 2025-12-29

**Authors:** Calvin Young, Chantelle Lachance, Allison Gates, Ana Komparic, Angie Hamson, Bernice Tsoi, Caitlyn Ford, Renata Axler, Joanne Kim, Chris Kamel, Laura Weeks

## Abstract

**Introduction:**

Canada’s Drug Agency (CDA-AMC) conducted a health technology assessment of RapidAI for detecting ischemic stroke and hemorrhagic stroke to test and learn from its first comprehensive assessment of an artificial intelligence (AI)-enabled health technology.

**Methods:**

The assessment included a review evaluating the effectiveness, accuracy, and cost-effectiveness of RapidAI for detecting ischemic and hemorrhagic stroke, alongside an implementation review capturing digital infrastructure considerations. Ethics and equity considerations were integrated throughout, informed by literature, patient engagement, and expert input. Checklists and other AI or digital health tools were applied. The Health Technology Expert Review Panel (HTERP), an advisory body to CDA-AMC, reviewed the evidence and developed recommendations on the appropriate use of RapidAI for stroke detection, considering the following domains: unmet clinical need, clinical value, economic considerations, impacts on health systems, and distinct social and ethical considerations.

**Results:**

Patient input highlighted speed and accuracy in stroke diagnosis. Low certainty clinical evidence suggested that using the AI functionalities of RapidAI to assist diagnoses may result in clinically important time reductions. Its effects on other clinical outcomes were very uncertain. Ethical and equity considerations have implications across the technology life cycle when using RapidAI for detecting stroke; however, little relevant information was identified from the literature. We found no relevant economic evaluations. The implementation review identified key considerations for AI-enabled health technologies for decision-makers. Given the evidence gaps and uncertainty, HTERP could not recommend for or against the use of RapidAI for stroke detection.

**Conclusions:**

Our appraisal and deliberative processes identified evidence limitations that may be common across many AI-enabled health technologies, identifying challenges that need to be addressed in their evaluation. Based on this experience, for AI evaluations CDA-AMC plans to add AI-specific implementation and other considerations to its evidence reviews and to consider a broader range of information sources.

